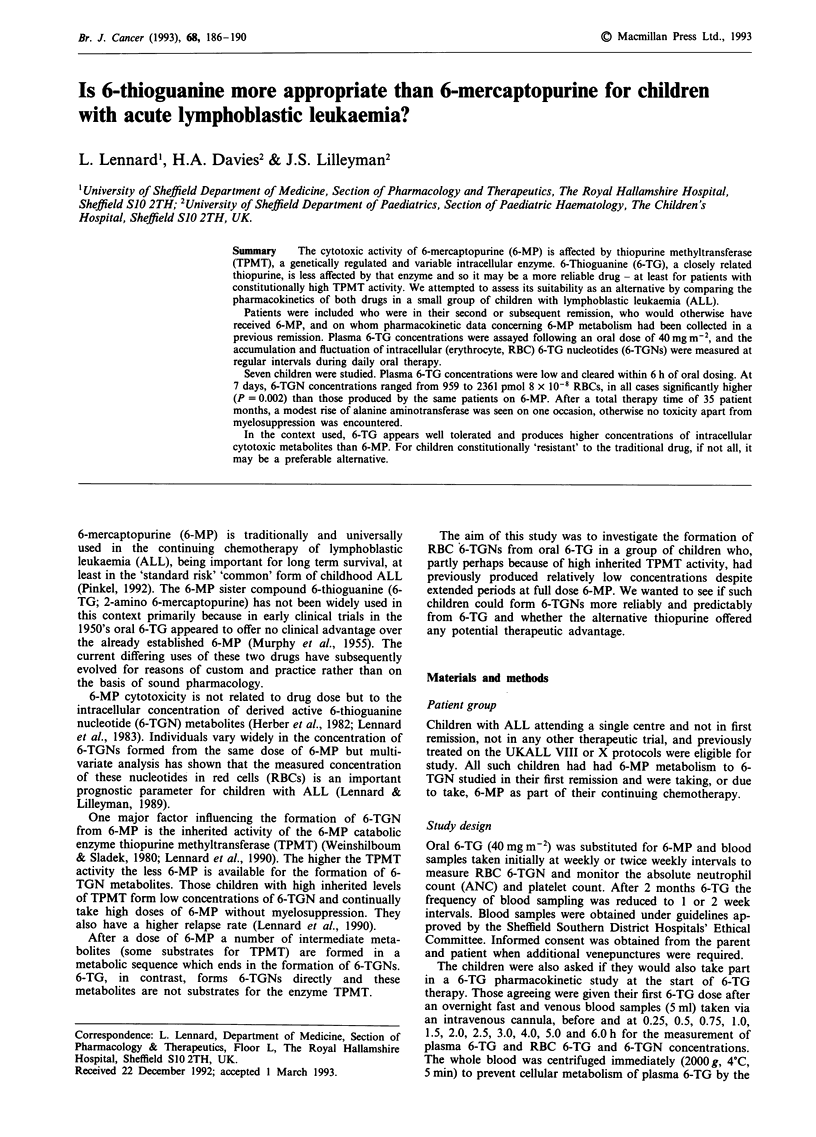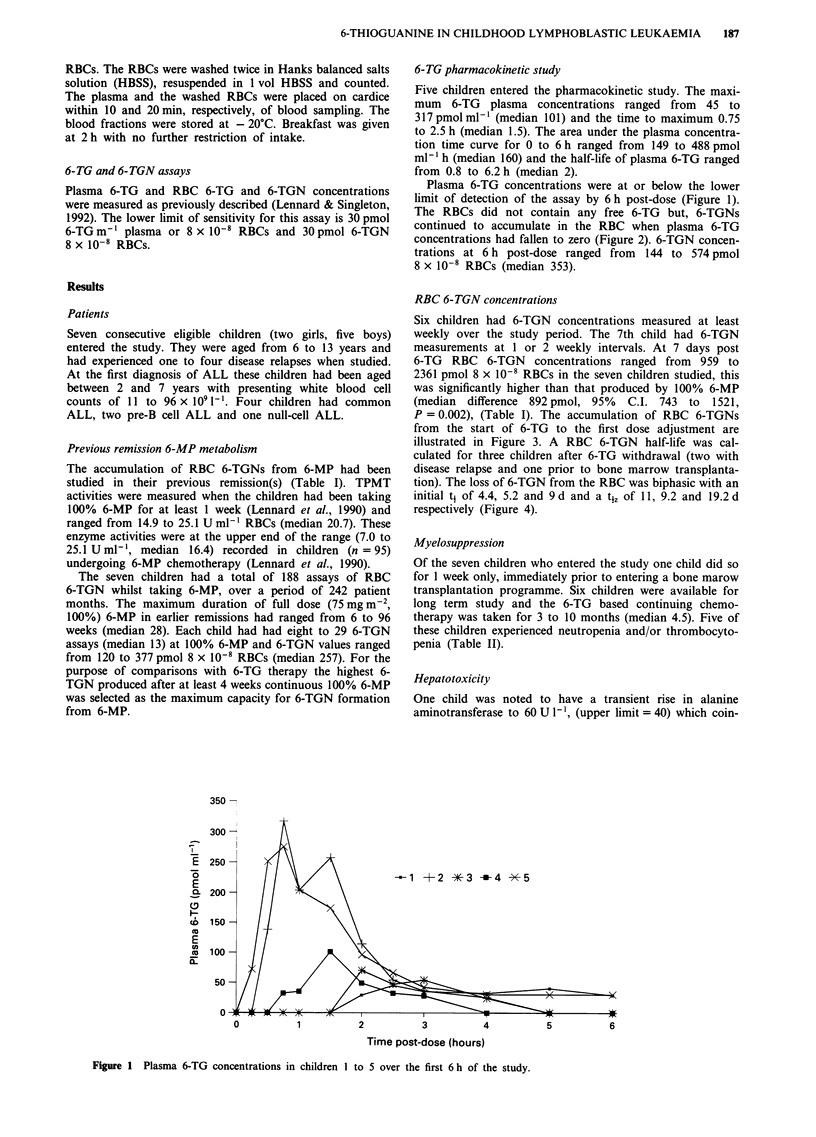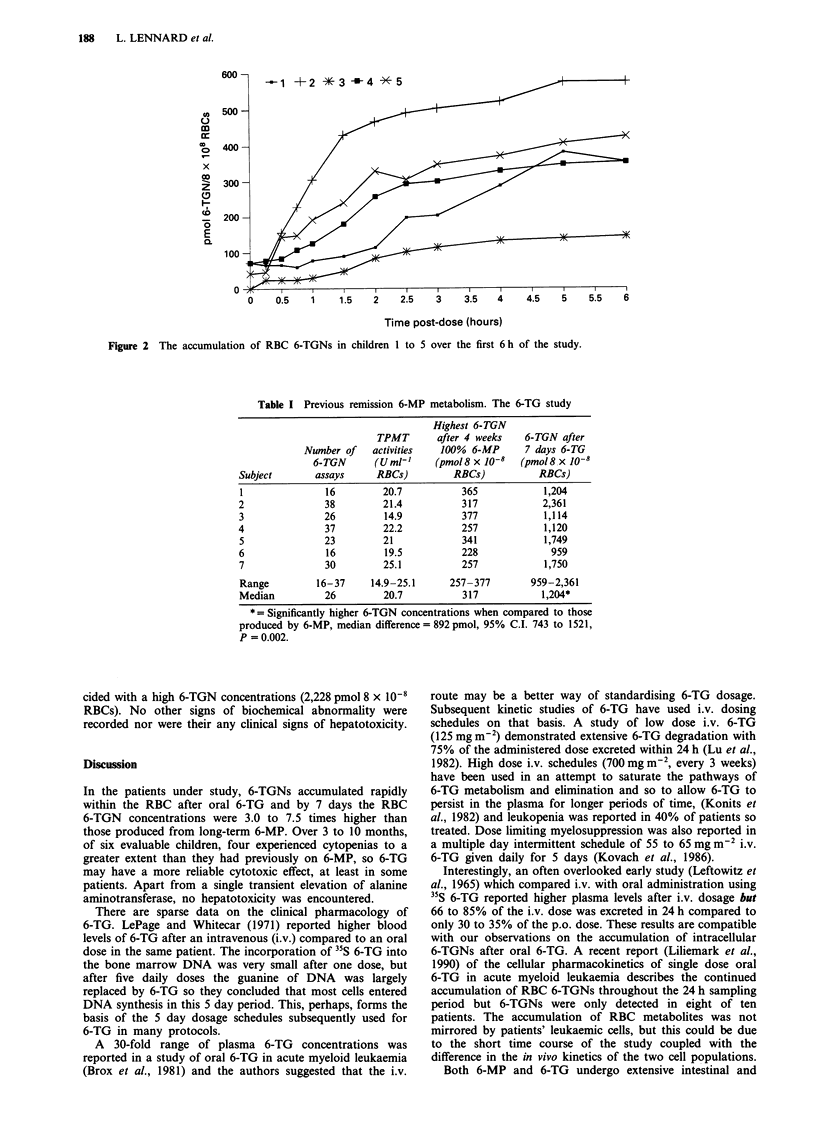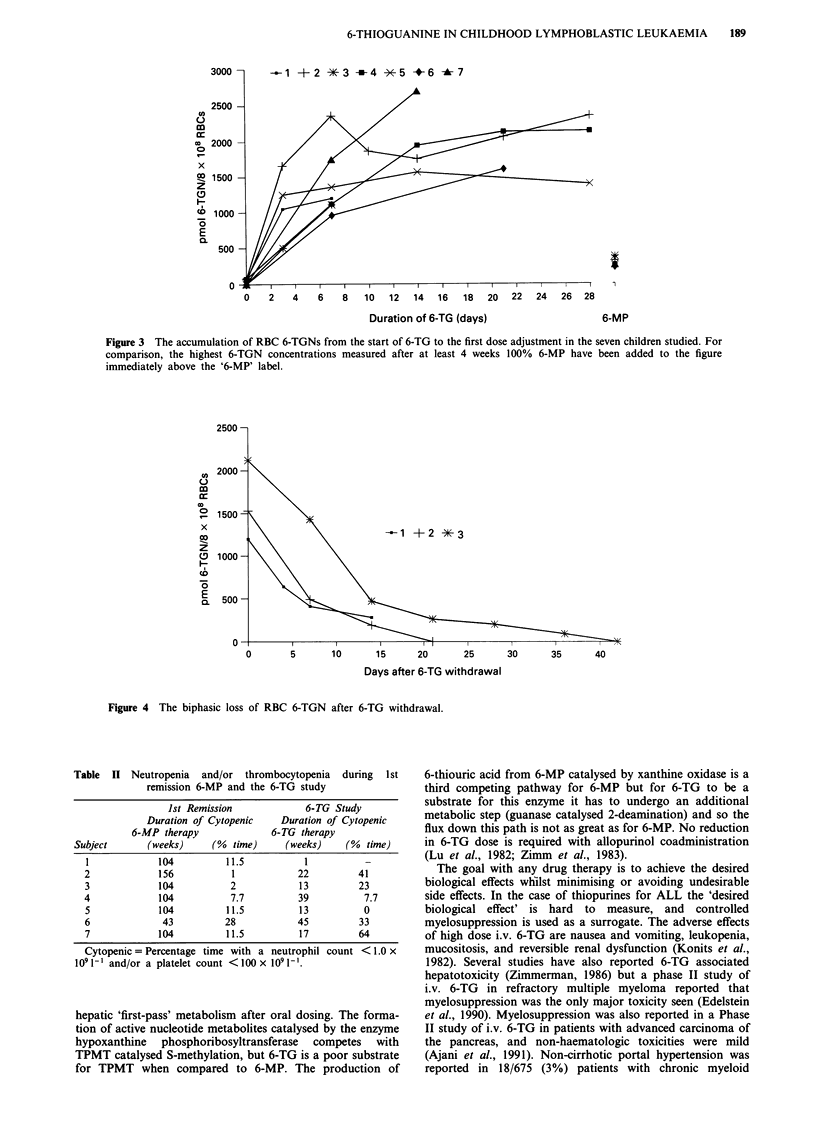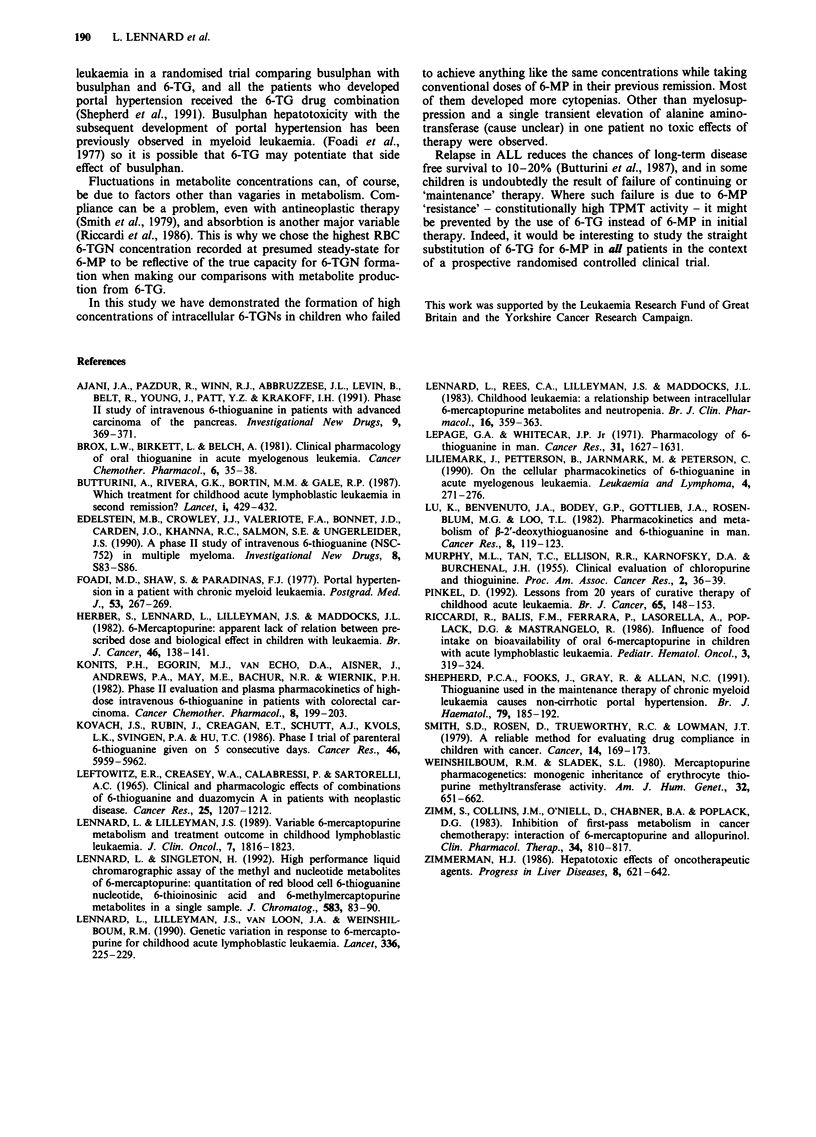# Is 6-thioguanine more appropriate than 6-mercaptopurine for children with acute lymphoblastic leukaemia?

**DOI:** 10.1038/bjc.1993.311

**Published:** 1993-07

**Authors:** L. Lennard, H. A. Davies, J. S. Lilleyman

**Affiliations:** University of Sheffield Department of Medicine, Royal Hallamshire Hospital, UK.

## Abstract

The cytotoxic activity of 6-mercaptopurine (6-MP) is affected by thiopurine methyltransferase (TPMT), a genetically regulated and variable intracellular enzyme. 6-Thioguanine (6-TG), a closely related thiopurine, is less affected by that enzyme and so it may be a more reliable drug-at least for patients with constitutionally high TPMT activity. We attempted to assess its suitability as an alternative by comparing the pharmacokinetics of both drugs in a small group of children with lymphoblastic leukaemia (ALL). Patients were included who were in their second or subsequent remission, who would otherwise have received 6-MP, and on whom pharmacokinetic data concerning 6-MP metabolism had been collected in a previous remission. Plasma 6-TG concentrations were assayed following an oral dose of 40 mg m-2, and the accumulation and fluctuation of intracellular (erythrocyte, RBC) 6-TG nucleotides (6-TGNs) were measured at regular intervals during daily oral therapy. Seven children were studied. Plasma 6-TG concentrations were low and cleared within 6 h of oral dosing. At 7 days, 6-TGN concentrations ranged from 959 to 2361 pmol 8 x 10(-8) RBCs, in all cases significantly higher (P = 0.002) than those produced by the same patients on 6-MP. After a total therapy time of 35 patient months, a modest rise of alanine aminotransferase was seen on one occasion, otherwise no toxicity apart from myelosuppression was encountered. In the context used, 6-TG appears well tolerated and produces higher concentrations of intracellular cytotoxic metabolites than 6-MP. For children constitutionally 'resistant' to the traditional drug, if not all, it may be a preferable alternative.


Br. J. Cancer (1993), 68, 186–190 © Macmillan Press Ltd., 1993

### Is 6-thioguanine more appropriate than 6-mercaptopurine for children with acute lymphoblastic leukaemia?

L. Lennard[1], H.A. Davies[2] & J.S. Lilleyman[2]

[1]*University of Sheffield Department of Medicine, Section of Pharmacology and Therapeutics, The Royal Hallamshire Hospital, Sheffield S10 2TH;* [2]*University of Sheffield Department of Paediatrics, Section of Paediatric Haematology, The Children's Hospital, Sheffield S10 2TH, UK.*

**Summary** The cytotoxic activity of 6-mercaptopurine (6-MP) is affected by thiopurine methyltransferase (TPMT), a genetically regulated and variable intracellular enzyme. 6-Thioguanine (6-TG), a closely related thiopurine, is less affected by that enzyme and so it may be a more reliable drug – at least for patients with constitutionally high TPMT activity. We attempted to assess its suitability as an alternative by comparing the pharmacokinetics of both drugs in a small group of children with lymphoblastic leukaemia (ALL).

Patients were included who were in their second or subsequent remission, who would otherwise have received 6-MP, and on whom pharmacokinetic data concerning 6-MP metabolism had been collected in a previous remission. Plasma 6-TG concentrations were assayed following an oral dose of 40 mg $m^{-2}$, and the accumulation and fluctuation of intracellular (erythrocyte, RBC) 6-TG nucleotides (6-TGNs) were measured at regular intervals during daily oral therapy.

Seven children were studied. Plasma 6-TG concentrations were low and cleared within 6 h of oral dosing. At 7 days, 6-TGN concentrations ranged from 959 to 2361 pmol $8 \times 10^{-8}$ RBCs, in all cases significantly higher ($P = 0.002$) than those produced by the same patients on 6-MP. After a total therapy time of 35 patient months, a modest rise of alanine aminotransferase was seen on one occasion, otherwise no toxicity apart from myelosuppression was encountered.

In the context used, 6-TG appears well tolerated and produces higher concentrations of intracellular cytotoxic metabolites than 6-MP. For children constitutionally 'resistant' to the traditional drug, if not all, it may be a preferable alternative.

6-mercaptopurine (6-MP) is traditionally and universally used in the continuing chemotherapy of lymphoblastic leukaemia (ALL), being important for long term survival, at least in the 'standard risk' 'common' form of childhood ALL (Pinkel, 1992). The 6-MP sister compound 6-thioguanine (6-TG; 2-amino 6-mercaptopurine) has not been widely used in this context primarily because in early clinical trials in the 1950's oral 6-TG appeared to offer no clinical advantage over the already established 6-MP (Murphy *et al.*, 1955). The current differing uses of these two drugs have subsequently evolved for reasons of custom and practice rather than on the basis of sound pharmacology.

6-MP cytotoxicity is not related to drug dose but to the intracellular concentration of derived active 6-thioguanine nucleotide (6-TGN) metabolites (Herber *et al.*, 1982; Lennard *et al.*, 1983). Individuals vary widely in the concentration of 6-TGNs formed from the same dose of 6-MP but multivariate analysis has shown that the measured concentration of these nucleotides in red cells (RBCs) is an important prognostic parameter for children with ALL (Lennard & Lilleyman, 1989).

One major factor influencing the formation of 6-TGN from 6-MP is the inherited activity of the 6-MP catabolic enzyme thiopurine methyltransferase (TPMT) (Weinshilboum & Sladek, 1980; Lennard *et al.*, 1990). The higher the TPMT activity the less 6-MP is available for the formation of 6-TGN metabolites. Those children with high inherited levels of TPMT form low concentrations of 6-TGN and continually take high doses of 6-MP without myelosuppression. They also have a higher relapse rate (Lennard *et al.*, 1990).

After a dose of 6-MP a number of intermediate metabolites (some substrates for TPMT) are formed in a metabolic sequence which ends in the formation of 6-TGNs. 6-TG, in contrast, forms 6-TGNs directly and these metabolites are not substrates for the enzyme TPMT.

Correspondence: L. Lennard, Department of Medicine, Section of Pharmacology & Therapeutics, Floor L, The Royal Hallamshire Hospital, Sheffield S10 2TH, UK.

Received 22 December 1992; accepted 1 March 1993.

The aim of this study was to investigate the formation of RBC 6-TGNs from oral 6-TG in a group of children who, partly perhaps because of high inherited TPMT activity, had previously produced relatively low concentrations despite extended periods at full dose 6-MP. We wanted to see if such children could form 6-TGNs more reliably and predictably from 6-TG and whether the alternative thiopurine offered any potential therapeutic advantage.

### Materials and methods

### *Patient group*

Children with ALL attending a single centre and not in first remission, not in any other therapeutic trial, and previously treated on the UKALL VIII or X protocols were eligible for study. All such children had had 6-MP metabolism to 6-TGN studied in their first remission and were taking, or due to take, 6-MP as part of their continuing chemotherapy.

### *Study design*

Oral 6-TG (40 mg $m^{-2}$) was substituted for 6-MP and blood samples taken initially at weekly or twice weekly intervals to measure RBC 6-TGN and monitor the absolute neutrophil count (ANC) and platelet count. After 2 months 6-TG the frequency of blood sampling was reduced to 1 or 2 week intervals. Blood samples were obtained under guidelines approved by the Sheffield Southern District Hospitals' Ethical Committee. Informed consent was obtained from the parent and patient when additional venepunctures were required.

The children were also asked if they would also take part in a 6-TG pharmacokinetic study at the start of 6-TG therapy. Those agreeing were given their first 6-TG dose after an overnight fast and venous blood samples (5 ml) taken via an intravenous cannula, before and at 0.25, 0.5, 0.75, 1.0, 1.5, 2.0, 2.5, 3.0, 4.0, 5.0 and 6.0 h for the measurement of plasma 6-TG and RBC 6-TG and 6-TGN concentrations. The whole blood was centrifuged immediately (2000 *g*, 4°C, 5 min) to prevent cellular metabolism of plasma 6-TG by the

RBCs. The RBCs were washed twice in Hanks balanced salts solution (HBSS), resuspended in 1 vol HBSS and counted. The plasma and the washed RBCs were placed on cardice within 10 and 20 min, respectively, of blood sampling. The blood fractions were stored at − 20°C. Breakfast was given at 2 h with no further restriction of intake.

*6-TG and 6-TGN assays*

Plasma 6-TG and RBC 6-TG and 6-TGN concentrations were measured as previously described (Lennard & Singleton, 1992). The lower limit of sensitivity for this assay is 30 pmol 6-TG $m^{-1}$ plasma or $8 \times 10^{-8}$ RBCs and 30 pmol 6-TGN $8 \times 10^{-8}$ RBCs.

### Results

*Patients*

Seven consecutive eligible children (two girls, five boys) entered the study. They were aged from 6 to 13 years and had experienced one to four disease relapses when studied. At the first diagnosis of ALL these children had been aged between 2 and 7 years with presenting white blood cell counts of 11 to $96 \times 10^9 l^{-1}$. Four children had common ALL, two pre-B cell ALL and one null-cell ALL.

*Previous remission 6-MP metabolism*

The accumulation of RBC 6-TGNs from 6-MP had been studied in their previous remission(s) (Table I). TPMT activities were measured when the children had been taking 100% 6-MP for at least 1 week (Lennard *et al.*, 1990) and ranged from 14.9 to 25.1 U $ml^{-1}$ RBCs (median 20.7). These enzyme activities were at the upper end of the range (7.0 to 25.1 U $ml^{-1}$, median 16.4) recorded in children ($n = 95$) undergoing 6-MP chemotherapy (Lennard *et al.*, 1990).

The seven children had a total of 188 assays of RBC 6-TGN whilst taking 6-MP, over a period of 242 patient months. The maximum duration of full dose (75 mg $m^{-2}$, 100%) 6-MP in earlier remissions had ranged from 6 to 96 weeks (median 28). Each child had had eight to 29 6-TGN assays (median 13) at 100% 6-MP and 6-TGN values ranged from 120 to 377 pmol $8 \times 10^{-8}$ RBCs (median 257). For the purpose of comparisons with 6-TG therapy the highest 6-TGN produced after at least 4 weeks continuous 100% 6-MP was selected as the maximum capacity for 6-TGN formation from 6-MP.

*6-TG pharmacokinetic study*

Five children entered the pharmacokinetic study. The maximum 6-TG plasma concentrations ranged from 45 to 317 pmol $ml^{-1}$ (median 101) and the time to maximum 0.75 to 2.5 h (median 1.5). The area under the plasma concentration time curve for 0 to 6 h ranged from 149 to 488 pmol $ml^{-1}$ h (median 160) and the half-life of plasma 6-TG ranged from 0.8 to 6.2 h (median 2).

Plasma 6-TG concentrations were at or below the lower limit of detection of the assay by 6 h post-dose (Figure 1). The RBCs did not contain any free 6-TG but, 6-TGNs continued to accumulate in the RBC when plasma 6-TG concentrations had fallen to zero (Figure 2). 6-TGN concentrations at 6 h post-dose ranged from 144 to 574 pmol $8 \times 10^{-8}$ RBCs (median 353).

*RBC 6-TGN concentrations*

Six children had 6-TGN concentrations measured at least weekly over the study period. The 7th child had 6-TGN measurements at 1 or 2 weekly intervals. At 7 days post 6-TG RBC 6-TGN concentrations ranged from 959 to 2361 pmol $8 \times 10^{-8}$ RBCs in the seven children studied, this was significantly higher than that produced by 100% 6-MP (median difference 892 pmol, 95% C.I. 743 to 1521, $P = 0.002$), (Table I). The accumulation of RBC 6-TGNs from the start of 6-TG to the first dose adjustment are illustrated in Figure 3. A RBC 6-TGN half-life was calculated for three children after 6-TG withdrawal (two with disease relapse and one prior to bone marrow transplantation). The loss of 6-TGN from the RBC was biphasic with an initial $t_{\frac{1}{2}}$ of 4.4, 5.2 and 9 d and a $t_{\frac{1}{2}z}$ of 11, 9.2 and 19.2 d respectively (Figure 4).

*Myelosuppression*

Of the seven children who entered the study one child did so for 1 week only, immediately prior to entering a bone marow transplantation programme. Six children were available for long term study and the 6-TG based continuing chemotherapy was taken for 3 to 10 months (median 4.5). Five of these children experienced neutropenia and/or thrombocytopenia (Table II).

*Hepatotoxicity*

One child was noted to have a transient rise in alanine aminotransferase to 60 U $l^{-1}$, (upper limit = 40) which coin-

**Figure 1** Plasma 6-TG concentrations in children 1 to 5 over the first 6 h of the study.

**Figure 2** The accumulation of RBC 6-TGNs in children 1 to 5 over the first 6 h of the study.

**Table I** Previous remission 6-MP metabolism. The 6-TG study

| *Subject* | *Number of 6-TGN assays* | *TPMT activities ($U\,ml^{-1}$ RBCs)* | *Highest 6-TGN after 4 weeks 100% 6-MP (pmol $8 \times 10^{-8}$ RBCs)* | *6-TGN after 7 days 6-TG (pmol $8 \times 10^{-8}$ RBCs)* |
|---|---|---|---|---|
| 1 | 16 | 20.7 | 365 | 1,204 |
| 2 | 38 | 21.4 | 317 | 2,361 |
| 3 | 26 | 14.9 | 377 | 1,114 |
| 4 | 37 | 22.2 | 257 | 1,120 |
| 5 | 23 | 21 | 341 | 1,749 |
| 6 | 16 | 19.5 | 228 | 959 |
| 7 | 30 | 25.1 | 257 | 1,750 |
| Range | 16–37 | 14.9–25.1 | 257–377 | 959–2,361 |
| Median | 26 | 20.7 | 317 | 1,204* |

* = Significantly higher 6-TGN concentrations when compared to those produced by 6-MP, median difference = 892 pmol, 95% C.I. 743 to 1521, $P = 0.002$.

cided with a high 6-TGN concentrations (2,228 pmol $8 \times 10^{-8}$ RBCs). No other signs of biochemical abnormality were recorded nor were their any clinical signs of hepatotoxicity.

### Discussion

In the patients under study, 6-TGNs accumulated rapidly within the RBC after oral 6-TG and by 7 days the RBC 6-TGN concentrations were 3.0 to 7.5 times higher than those produced from long-term 6-MP. Over 3 to 10 months, of six evaluable children, four experienced cytopenias to a greater extent than they had previously on 6-MP, so 6-TG may have a more reliable cytotoxic effect, at least in some patients. Apart from a single transient elevation of alanine aminotransferase, no hepatotoxicity was encountered.

There are sparse data on the clinical pharmacology of 6-TG. LePage and Whitecar (1971) reported higher blood levels of 6-TG after an intravenous (i.v.) compared to an oral dose in the same patient. The incorporation of $^{35}S$ 6-TG into the bone marrow DNA was very small after one dose, but after five daily doses the guanine of DNA was largely replaced by 6-TG so they concluded that most cells entered DNA synthesis in this 5 day period. This, perhaps, forms the basis of the 5 day dosage schedules subsequently used for 6-TG in many protocols.

A 30-fold range of plasma 6-TG concentrations was reported in a study of oral 6-TG in acute myeloid leukaemia (Brox *et al.*, 1981) and the authors suggested that the i.v. route may be a better way of standardising 6-TG dosage. Subsequent kinetic studies of 6-TG have used i.v. dosing schedules on that basis. A study of low dose i.v. 6-TG ($125\,mg\,m^{-2}$) demonstrated extensive 6-TG degradation with 75% of the administered dose excreted within 24 h (Lu *et al.*, 1982). High dose i.v. schedules ($700\,mg\,m^{-2}$, every 3 weeks) have been used in an attempt to saturate the pathways of 6-TG metabolism and elimination and so to allow 6-TG to persist in the plasma for longer periods of time, (Konits *et al.*, 1982) and leukopenia was reported in 40% of patients so treated. Dose limiting myelosuppression was also reported in a multiple day intermittent schedule of 55 to $65\,mg\,m^{-2}$ i.v. 6-TG given daily for 5 days (Kovach *et al.*, 1986).

Interestingly, an often overlooked early study (Leftowitz *et al.*, 1965) which compared i.v. with oral administration using $^{35}S$ 6-TG reported higher plasma levels after i.v. dosage **but** 66 to 85% of the i.v. dose was excreted in 24 h compared to only 30 to 35% of the p.o. dose. These results are compatible with our observations on the accumulation of intracellular 6-TGNs after oral 6-TG. A recent report (Liliemark *et al.*, 1990) of the cellular pharmacokinetics of single dose oral 6-TG in acute myeloid leukaemia describes the continued accumulation of RBC 6-TGNs throughout the 24 h sampling period but 6-TGNs were only detected in eight of ten patients. The accumulation of RBC metabolites was not mirrored by patients' leukaemic cells, but this could be due to the short time course of the study coupled with the difference in the *in vivo* kinetics of the two cell populations.

Both 6-MP and 6-TG undergo extensive intestinal and

**Figure 3** The accumulation of RBC 6-TGNs from the start of 6-TG to the first dose adjustment in the seven children studied. For comparison, the highest 6-TGN concentrations measured after at least 4 weeks 100% 6-MP have been added to the figure immediately above the '6-MP' label.

**Figure 4** The biphasic loss of RBC 6-TGN after 6-TG withdrawal.

**Table II** Neutropenia and/or thrombocytopenia during 1st remission 6-MP and the 6-TG study

| Subject | *1st Remission* Duration of 6-MP therapy (weeks) | *1st Remission* Cytopenic (% time) | *6-TG Study* Duration of 6-TG therapy (weeks) | *6-TG Study* Cytopenic (% time) |
|---|---|---|---|---|
| 1 | 104 | 11.5 | 1 | – |
| 2 | 156 | 1 | 22 | 41 |
| 3 | 104 | 2 | 13 | 23 |
| 4 | 104 | 7.7 | 39 | 7.7 |
| 5 | 104 | 11.5 | 13 | 0 |
| 6 | 43 | 28 | 45 | 33 |
| 7 | 104 | 11.5 | 17 | 64 |

Cytopenic = Percentage time with a neutrophil count $< 1.0 \times 10^9 l^{-1}$ and/or a platelet count $< 100 \times 10^9 l^{-1}$.

hepatic 'first-pass' metabolism after oral dosing. The formation of active nucleotide metabolites catalysed by the enzyme hypoxanthine phosphoribosyltransferase competes with TPMT catalysed S-methylation, but 6-TG is a poor substrate for TPMT when compared to 6-MP. The production of 6-thiouric acid from 6-MP catalysed by xanthine oxidase is a third competing pathway for 6-MP but for 6-TG to be a substrate for this enzyme it has to undergo an additional metabolic step (guanase catalysed 2-deamination) and so the flux down this path is not as great as for 6-MP. No reduction in 6-TG dose is required with allopurinol coadministration (Lu *et al.*, 1982; Zimm *et al.*, 1983).

The goal with any drug therapy is to achieve the desired biological effects whilst minimising or avoiding undesirable side effects. In the case of thiopurines for ALL the 'desired biological effect' is hard to measure, and controlled myelosuppression is used as a surrogate. The adverse effects of high dose i.v. 6-TG are nausea and vomiting, leukopenia, mucositosis, and reversible renal dysfunction (Konits *et al.*, 1982). Several studies have also reported 6-TG associated hepatotoxicity (Zimmerman, 1986) but a phase II study of i.v. 6-TG in refractory multiple myeloma reported that myelosuppression was the only major toxicity seen (Edelstein *et al.*, 1990). Myelosuppression was also reported in a Phase II study of i.v. 6-TG in patients with advanced carcinoma of the pancreas, and non-haematologic toxicities were mild (Ajani *et al.*, 1991). Non-cirrhotic portal hypertension was reported in 18/675 (3%) patients with chronic myeloid leukaemia in a randomised trial comparing busulphan with busulphan and 6-TG, and all the patients who developed portal hypertension received the 6-TG drug combination (Shepherd *et al.*, 1991). Busulphan hepatotoxicity with the subsequent development of portal hypertension has been previously observed in myeloid leukaemia. (Foadi *et al.*, 1977) so it is possible that 6-TG may potentiate that side effect of busulphan.

Fluctuations in metabolite concentrations can, of course, be due to factors other than vagaries in metabolism. Compliance can be a problem, even with antineoplastic therapy (Smith *et al.*, 1979), and absorbtion is another major variable (Riccardi *et al.*, 1986). This is why we chose the highest RBC 6-TGN concentration recorded at presumed steady-state for 6-MP to be reflective of the true capacity for 6-TGN formation when making our comparisons with metabolite production from 6-TG.

In this study we have demonstrated the formation of high concentrations of intracellular 6-TGNs in children who failed to achieve anything like the same concentrations while taking conventional doses of 6-MP in their previous remission. Most of them developed more cytopenias. Other than myelosuppression and a single transient elevation of alanine aminotransferase (cause unclear) in one patient no toxic effects of therapy were observed.

Relapse in ALL reduces the chances of long-term disease free survival to 10–20% (Butturini *et al.*, 1987), and in some children is undoubtedly the result of failure of continuing or 'maintenance' therapy. Where such failure is due to 6-MP 'resistance' – constitutionally high TPMT activity – it might be prevented by the use of 6-TG instead of 6-MP in initial therapy. Indeed, it would be interesting to study the straight substitution of 6-TG for 6-MP in ***all*** patients in the context of a prospective randomised controlled clinical trial.

This work was supported by the Leukaemia Research Fund of Great Britain and the Yorkshire Cancer Research Campaign.

### References

AJANI, J.A., PAZDUR, R., WINN, R.J., ABBRUZZESE, J.L., LEVIN, B., BELT, R., YOUNG, J., PATT, Y.Z. & KRAKOFF, I.H. (1991). Phase II study of intravenous 6-thioguanine in patients with advanced carcinoma of the pancreas. *Investigational New Drugs*, **9**, 369–371.

BROX, L.W., BIRKETT, L. & BELCH, A. (1981). Clinical pharmacology of oral thioguanine in acute myelogenous leukemia. *Cancer Chemother. Pharmacol.*, **6**, 35–38.

BUTTURINI, A., RIVERA, G.K., BORTIN, M.M. & GALE, R.P. (1987). Which treatment for childhood acute lymphoblastic leukaemia in second remission? *Lancet*, **i**, 429–432.

EDELSTEIN, M.B., CROWLEY, J.J., VALERIOTE, F.A., BONNET, J.D., CARDEN, J.O., KHANNA, R.C., SALMON, S.E. & UNGERLEIDER, J.S. (1990). A phase II study of intravenous 6-thioguanine (NSC-752) in multiple myeloma. *Investigational New Drugs*, **8**, S83–S86.

FOADI, M.D., SHAW, S. & PARADINAS, F.J. (1977). Portal hypertension in a patient with chronic myeloid leukaemia. *Postgrad. Med. J.*, **53**, 267–269.

HERBER, S., LENNARD, L., LILLEYMAN, J.S. & MADDOCKS, J.L. (1982). 6-Mercaptopurine: apparent lack of relation between prescribed dose and biological effect in children with leukaemia. *Br. J. Cancer*, **46**, 138–141.

KONITS, P.H., EGORIN, M.J., VAN ECHO, D.A., AISNER, J., ANDREWS, P.A., MAY, M.E., BACHUR, N.R. & WIERNIK, P.H. (1982). Phase II evaluation and plasma pharmacokinetics of high-dose intravenous 6-thioguanine in patients with colorectal carcinoma. *Cancer Chemother. Pharmacol.*, **8**, 199–203.

KOVACH, J.S., RUBIN, J., CREAGAN, E.T., SCHUTT, A.J., KVOLS, L.K., SVINGEN, P.A. & HU, T.C. (1986). Phase I trial of parenteral 6-thioguanine given on 5 consecutive days. *Cancer Res.*, **46**, 5959–5962.

LEFTOWITZ, E.R., CREASEY, W.A., CALABRESSI, P. & SARTORELLI, A.C. (1965). Clinical and pharmacologic effects of combinations of 6-thioguanine and duazomycin A in patients with neoplastic disease. *Cancer Res.*, **25**, 1207–1212.

LENNARD, L. & LILLEYMAN, J.S. (1989). Variable 6-mercaptopurine metabolism and treatment outcome in childhood lymphoblastic leukaemia. *J. Clin. Oncol.*, **7**, 1816–1823.

LENNARD, L. & SINGLETON, H. (1992). High performance liquid chromarographic assay of the methyl and nucleotide metabolites of 6-mercaptopurine: quantitation of red blood cell 6-thioguanine nucleotide, 6-thioinosinic acid and 6-methylmercaptopurine metabolites in a single sample. *J. Chromatog.*, **583**, 83–90.

LENNARD, L., LILLEYMAN, J.S., VAN LOON, J.A. & WEINSHILBOUM, R.M. (1990). Genetic variation in response to 6-mercaptopurine for childhood acute lymphoblastic leukaemia. *Lancet*, **336**, 225–229.

LENNARD, L., REES, C.A., LILLEYMAN, J.S. & MADDOCKS, J.L. (1983). Childhood leukaemia: a relationship between intracellular 6-mercaptopurine metabolites and neutropenia. *Br. J. Clin. Pharmacol.*, **16**, 359–363.

LEPAGE, G.A. & WHITECAR, J.P. Jr (1971). Pharmacology of 6-thioguanine in man. *Cancer Res.*, **31**, 1627–1631.

LILIEMARK, J., PETTERSON, B., JARNMARK, M. & PETERSON, C. (1990). On the cellular pharmacokinetics of 6-thioguanine in acute myelogenous leukaemia. *Leukaemia and Lymphoma*, **4**, 271–276.

LU, K., BENVENUTO, J.A., BODEY, G.P., GOTTLIEB, J.A., ROSENBLUM, M.G. & LOO, T.L. (1982). Pharmacokinetics and metabolism of β-2′-deoxythioguanosine and 6-thioguanine in man. *Cancer Res.*, **8**, 119–123.

MURPHY, M.L., TAN, T.C., ELLISON, R.R., KARNOFSKY, D.A. & BURCHENAL, J.H. (1955). Clinical evaluation of chloropurine and thioguinine. *Proc. Am. Assoc. Cancer Res.*, **2**, 36–39.

PINKEL, D. (1992). Lessons from 20 years of curative therapy of childhood acute leukaemia. *Br. J. Cancer*, **65**, 148–153.

RICCARDI, R., BALIS, F.M., FERRARA, P., LASORELLA, A., POPLACK, D.G. & MASTRANGELO, R. (1986). Influence of food intake on bioavailability of oral 6-mercaptopurine in children with acute lymphoblastic leukaemia. *Pediatr. Hematol. Oncol.*, **3**, 319–324.

SHEPHERD, P.C.A., FOOKS, J., GRAY, R. & ALLAN, N.C. (1991). Thioguanine used in the maintenance therapy of chronic myeloid leukaemia causes non-cirrhotic portal hypertension. *Br. J. Haematol.*, **79**, 185–192.

SMITH, S.D., ROSEN, D., TRUEWORTHY, R.C. & LOWMAN, J.T. (1979). A reliable method for evaluating drug compliance in children with cancer. *Cancer*, **14**, 169–173.

WEINSHILBOUM, R.M. & SLADEK, S.L. (1980). Mercaptopurine pharmacogenetics: monogenic inheritance of erythrocyte thiopurine methyltransferase activity. *Am. J. Hum. Genet.*, **32**, 651–662.

ZIMM, S., COLLINS, J.M., O'NIELL, D., CHABNER, B.A. & POPLACK, D.G. (1983). Inhibition of first-pass metabolism in cancer chemotherapy: interaction of 6-mercaptopurine and allopurinol. *Clin. Pharmacol. Therap.*, **34**, 810–817.

ZIMMERMAN, H.J. (1986). Hepatotoxic effects of oncotherapeutic agents. *Progress in Liver Diseases*, **8**, 621–642.